# Motor activation is modulated by visual experience during cyclic gait observation: A transcranial magnetic stimulation study

**DOI:** 10.1371/journal.pone.0228389

**Published:** 2020-01-28

**Authors:** Tomotaka Ito, Akio Tsubahara, Yoshiki Shiraga, Yosuke Yoshimura, Daisuke Kimura, Keita Suzuki, Kozo Hanayama

**Affiliations:** 1 Department of Rehabilitation, Faculty of Health Science and Technology, Kawasaki University of Medical Welfare, Kurashiki, Okayama, Japan; 2 Rehabilitation Center, Kawasaki Medical School Hospital, Kurashiki, Okayama, Japan; 3 Department of Environmental and Preventive Medicine, Graduate School of Medical Science, Kanazawa University, Kanazawa, Ishikawa, Japan; 4 Department of Rehabilitation Medicine, Kawasaki Medical School, Kurashiki, Okayama, Japan; University of Bern, SWITZERLAND

## Abstract

Transcranial magnetic stimulation (TMS) has been widely utilized to noninvasively explore the motor system during the observation of human movement. However, few studies have characterized motor cortex activity during periodic gait observation. Thus, this study examined the effects of an observer’s visual experience and/or intention to imitate on corticospinal excitability during the observation of another’s gait. Twenty-six healthy volunteers were included in this study and allocated to two different groups. Participants in the visual experience group had formal experience with gait observation (physical therapist training), while those in the control group did not. Motor-evoked potentials induced by TMS in the tibialis anterior and soleus muscles were measured as surrogates of corticospinal excitability. Participants were seated and, while resting, they observed a demonstrator’s gait or observed it with the intention to subsequently reproduce it. Compared with the resting state, cyclic gait observation led to significant corticospinal facilitation in the tibialis anterior and soleus muscles. However, this pattern of corticospinal facilitation in the measured muscles was not coupled to the pattern of crural muscle activity during actual gait and was independent of the step cycle. This motor cortex facilitation effect during gait observation was enhanced by the observer’s visual experience in a manner that was not step cycle-dependent, while the observer’s intent to imitate did not affect corticospinal excitatory input to either muscle. In addition, visual experience did not modulate corticospinal excitability in gait-related crural muscles. Our findings indicate that motor cortex activity during gait observation is not in line with the timing of muscle activity during gait execution and is modulated by an individual’s gait observation experience. These results suggest that visual experience acquired from repetitive gait observation may facilitate the motor system’s control on bipedal walking, but may not promote the learning of muscle activity patterns.

## Introduction

Action observation therapy (AOT) is a novel rehabilitation approach used to promote motor recovery in both neurological [1–4] and orthopedic pathological contexts [5,6]. It is usually used in combination with the execution of observed movements in a clinical setting; patients are instructed to carefully observe actions presented in a video clip or demonstrated by a therapist in order to later imitate these actions.

The neural basis of AOT is presumed to be the mirror neuron system [7,8]. Mirror neurons are a specific class of visuomotor neurons originally discovered in area F5 of the monkey premotor cortex [9]. An important feature of these neurons is that they are activated in response to both the observation and execution of movements [9–11]. As it is not possible to directly record the activity of single neurons in the human brain, noninvasive techniques, such as brain imaging and transcranial magnetic stimulation (TMS), have been utilized to identify the existence of the human mirror neuron system and to explore its properties [12].

Recent brain imaging studies using functional magnetic resonance imaging (fMRI) have shown that regions of the supplementary motor area, dorsal premotor cortex, inferior frontal gyrus, and inferior parietal lobule are activated during the observation of gait movement [13]. Furthermore, since cortical activation of the parieto-frontal areas has been detected using fMRI during both the observation and the execution of walking, the existence of a so-called observation/execution matching system [11,14] that automatically matches the observed action and retrieved motor behavior in the motor system has been confirmed during gait observation [15]. As a large-scale meta-analysis identified consistent cortical activation of the bilateral premotor, parietal, and sensorimotor networks, which are induced by action observation and movement execution [16], this matching of brain regions during both tasks appears to support the clinical efficacy of AOT as a rehabilitation approach for individuals with gait disturbances [1,4,6].

Whereas brain imaging can identify cortical areas that are activated in response to movement observation, TMS methods measure corticospinal excitability with relatively high temporal resolution [17]. Many previous studies have demonstrated that motor-evoked potentials (MEPs) elicited by TMS during action observation are tightly coupled to the muscles involved in the performance and temporal pattern of the observed action [18–29]. Such studies have suggested that this muscular and temporal observation/execution matching reflects the motor program encoding kinematic parameters of the observed actions. However, MEPs elicited in gait-related muscles are constantly augmented throughout the step cycle during cyclic gait observation and are not matched to the actual muscle activity during gait [30]. This response to TMS during gait observation is inconsistent with the responses exhibited during the observation of other movements. Thus, a better understanding of motor cortex activity during gait observation may help improve the recognition and implementation of AOT in clinical settings.

With regard to the factors affecting an observer’s cortical activation during action observation, fMRI studies have demonstrated that observed actions that belong to the motor repertoire of an observer are mapped onto the observer’s motor system [31]. Moreover, Calvo-Merion et al. [32] indicated that brain activity during action observation is strongly influenced by the observer’s motor repertoire, depending on both the visual knowledge and motor representation of the action [33]. Given that walking is an innate and automated movement in humans, it is unlikely that there are any individual differences in motor repertoires concerning gait; however, a previous TMS study reported that the visual experience gained by observing movements also affects the activation of the motor system [18]. Further, the intent to imitate may be another factor affecting an observer’s cortical activation during action observation. Indeed, several imaging studies have reported that observing a hand movement with the intention to subsequently imitate the movement produces greater brain activation than observation alone [34,35]. Additionally, larger increases in corticospinal excitability have been identified in response to the observation of a complex task with the intent to imitate compared to task observation alone [36]. However, to our knowledge, no studies examining the effects of an observer’s visual experience and/or intent to imitate on corticospinal excitability during gait observation have been published. Studies in this direction would be useful to better understand the neural activity that occurs in the human brain during periodic gait observation.

The main aim of the present study was to examine the effects that individual differences in visual experience have on motor cortex activity during gait observation. For this purpose, we evaluated TMS-induced corticospinal excitability during gait observation in experienced and non-experienced observers. We also used two different observation conditions, i.e., gait observation alone (passive observation [PO]) and gait observation with the intent to imitate (active observation [AO]), to examine the effects of the intent to imitate on corticospinal excitability during gait observation. Based on the findings of previous studies [18,36], we hypothesized that: (1) individuals with more visual experience would exhibit corticospinal excitability facilitation, (2) this facilitation pattern in measured muscles might be different from that observed in individuals without visual experience, and (3) an observer’s intention to subsequently reproduce movements would also facilitate corticospinal excitability during gait observation. Furthermore, since we presumed that (4) another potential factor that could affect corticospinal excitability during the two observation conditions might be differences in motor imagery ability between visually experienced and non-experienced observers, we also investigated the interaction effect of visual experience and observation condition on motor cortex activity.

## Materials and methods

### Participants

The study conformed to the principles of the Declaration of Helsinki, and the study protocol was approved by the Research Ethics Committee of Kawasaki University of Medical Welfare (approval number 16–096). All participants provided written informed consent. In order to determine the sample size, a statistical power analysis was conducted using the G*Power software package. Since we were mainly interested in the effects of an observer’s visual experience (factor A) or intention to imitate (factor B) on corticospinal excitability, the effect size for the analysis of factor A was set at f = 0.595 based on data from a previous between-groups TMS study [37], and that for factor B was set at f = 0.850 based on data from similar published TMS research [38], with an alpha of 0.05 and a power of 0.80. The minimum sample size necessary for this experiment was thus estimated to be n = 25. However, since in our previous study using TMS [39] we were unable to obtain measurable MEP data from the tibialis anterior (TA) and soleus (SOL) muscles simultaneously in three out of 11 (27.2%) participants, we recruited more than 32 participants, to compensate for potentially corrupted data. A total of 36 healthy volunteers (18 men and 18 women; mean age, 21.4 ± 0.8 years) without any neurological or psychiatric deficits participated in this study. Participants were divided into the visual experience (n = 17) and the control group (n = 19), according to their major field of study at university. Participants in the visual experience group were enrolled in a physical therapist training course, had experienced gait observation through approximately 20 weeks of clinical education, and had kinematic knowledge of human bipedal walking. Participants in the control group consisted of students of other university departments. Prior to participating in this study, we asked each individual in the control group whether they had visual experience with gait from lectures or clinical education, and checked their curriculum to ensure that they did not have any formal gait observation experience or kinematic knowledge of gait. The two groups were matched for numbers of participants, age, and proportion of men to women; the characteristics of each group are shown in Table 1. All participants were confirmed to be right-handed, as assessed with the Edinburgh Handedness Inventory [40].

**Table 1:** Characteristics of participants in the visual experience and control groups.

		Visual experience group (n = 13)	Control group (n = 13)
**Age (years), mean (SD)**		21.7 (0.6)	21.2 (0.8)
**Sex (male/female)**		6:7	6:7
**TMS stimulation intensity (%), mean (SD)**		56.5 (5.9)	56.2 (7.7)
**Raw MEP area in the baseline condition (mVms), mean (SE)**	**TA**	1.552 (0.343)	1.634 (0.262)
	**SOL**	0.453 (0.090)	0.530 (0.067)
**Gait imagery ability (%), mean (SD)**		53.5 (25.0)	48.7 (24.0)

SD, standard deviation; TMS, transcranial magnetic stimulation; MEP, motor-evoked potential; SE, standard error; TA, tibialis anterior; SOL, soleus.

### Experimental conditions

Throughout the experiment, participants were seated on a chair with their feet hanging above the floor. A treadmill (Sakai Medical Co. Ltd., Tokyo, Japan) was located at a distance of approximately 1 m from the observers (participants). Observers watched a person (demonstrator) walk on the treadmill from the right sagittal plane [30]. For all experiments, the same demonstrator walked on the treadmill at a speed of 2 km/h [30], with his cadence matched to the tempo of a metronome at 76 beats per minute. Since auditory stimuli are known to affect brain activation [41] and attention, the demonstrator listened to the metronome through wearable earphones to maintain his walking at a constant tempo.

MEPs were elicited by TMS prior to (baseline) and during gait observation. A total of three TMS measurements with a 3-min interval between measurements were conducted during the course of the experiment. During baseline measurements, the observers were instructed to relax their body completely and to look at the static treadmill (without the demonstrator). The following two conditions were used for gait observation measurements: PO trials, in which participants were instructed to closely observe the movements of the demonstrator’s lower legs, and AO trials, in which participants were instructed to observe the movement of the demonstrator’s lower legs with an intent to imitate the movement. To minimize any after-effects of the intent to imitate on subsequent measurements, i.e., to prevent participants from observing gait with the intent to imitate during PO trials, PO trials always preceded AO trials, consistent with the design of a prior study [36]. In addition, previous research on cerebral activity during gait imagery revealed that observers with good imagery ability show higher activation in the primary motor cortex than observers with poor imagery ability [42]. Since we assumed that some participants would mentally rehearse (imagine) the demonstrator’s gait movements during the AO trials, all participants were asked, after the completion of the TMS measurements for later analyses, whether they imagined the gait during the PO and AO trials (see below).

### Electromyography

EMG responses to TMS were recorded from the right TA and SOL muscles using disposable Ag/AgCl surface electrodes that were 10 mm in diameter (Blue Sensor P-00-S; Ambu Co., Ballerup, Denmark). Since these two muscles in the lower limbs are commonly used in TMS studies to assess the involvement of the motor cortex during actual gait [43–45] and motor cortex activity during gait observation [30], in accordance with these prior studies, the TA and SOL muscles were selected as target muscles. Bipolar electrode pairs were placed longitudinally over the muscle belly at an inter-electrode distance of 20 mm. A ground electrode was attached to the right lateral malleolus. Prior to electrode configuration, the skin was abraded with a skin preparation gel (Skin Pure; Nihon Kohden Co., Tokyo, Japan) and then cleaned with alcohol to reduce skin impedance. The EMG signals were amplified using a bio-amplifier (BA1008; TEAC Co., Tokyo, Japan) and stored on a personal computer. The sensitivity, time constant, and high-cutoff filter of the amplifier were set at 200 μV/0.5 V, 0.01 s, and 3 kHz, respectively.

### TMS procedure

Single-pulse TMS was delivered using a double-cone coil with an outer diameter of 110 mm, connected to a Magstim 200^2^ magnetic stimulator (Magstim Co., Dyfed, UK). After identification of the Cz point (with reference to the international 10–20 system [46]) and before the initial baseline measurement, the coil was moved over the scalp to detect the optimal leg region for eliciting MEP amplitudes in the right TA and SOL muscles. The coil was oriented so that the induced electric current in the brain would flow in the posterior-anterior direction. This position was marked on a tight-fitting swim cap, which was then fixed to the skin with surgical tape to ensure that the same location was stimulated during each TMS measurement. The resting motor threshold was determined while the TA and SOL muscles were at rest and defined as the minimum stimulus intensity necessary to induce an MEP with an amplitude of 100 μV for at least six out of 10 consecutive stimuli. The experimental stimulus intensity was 120% of the resting motor threshold. Stimulus intensity was expressed as a percentage of the maximum output of the stimulator.

### Stimulation protocol

**Fig 1:**
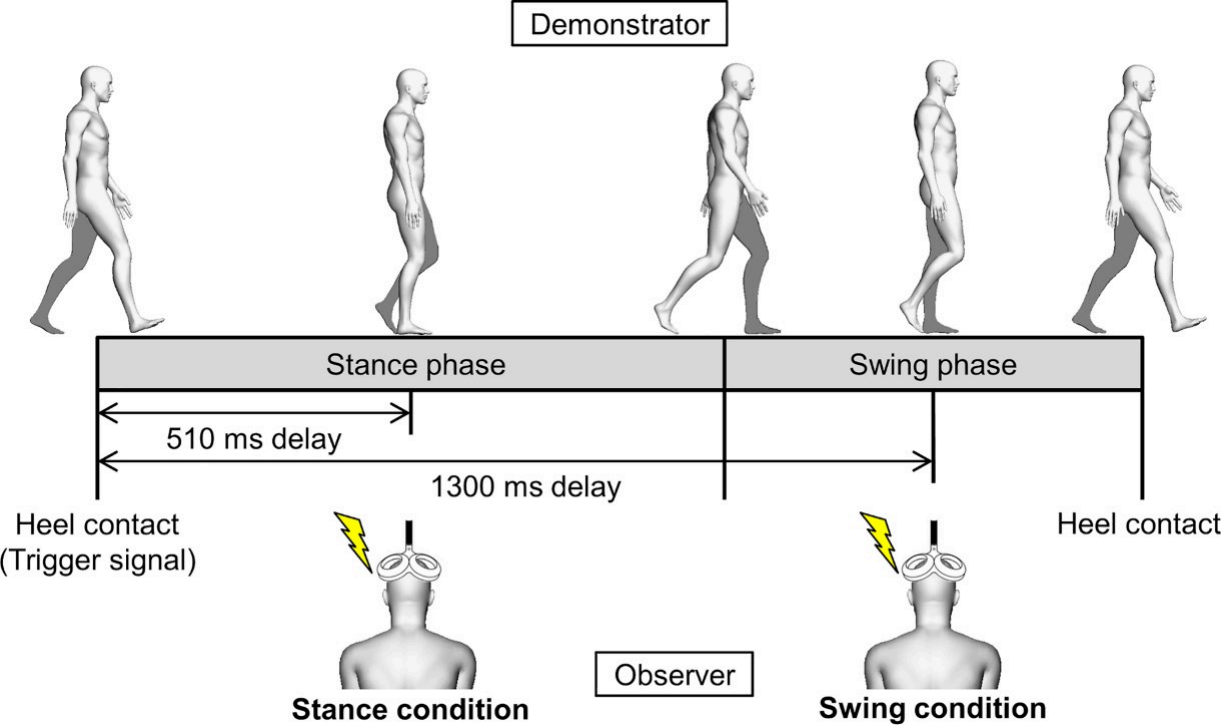
Stimulus timing during gait observation. The demonstrator’s right leg is shown in white and the left leg is shown in gray. The transcranial magnetic stimulation that was administered to the observer was triggered by the contact of the demonstrator’s right heel with the treadmill and was delivered at the midpoints of the stance phase (510 ms; stance condition) and swing phase (1300 ms; swing condition).

Eight MEPs were evoked by TMS during baseline, PO, and AO trials with an inter-stimulus interval of 7 s. In each trial, eight stimuli were delivered to the participants during the midpoints of the stance and swing phases of the demonstrator (stance condition and swing condition, respectively), in a pseudorandom order, using a pressure-sensitive foot switch attached to the demonstrator’s right heel. Therefore, a total of 16 MEPs were recorded in each trial. Prior to the experiment, the demonstrator’s average stance and swing phase times were measured using shoe-type plate sensors (Gait Coder MP-1000; Anima Co., Tokyo, Japan) while the demonstrator walked on the treadmill at 2 km/h at a tempo of 76 beats per minute indicated by a metronome. In accordance with this measurement, the stimulus delivery time was set at 510 ms in the stance phase, and at 1300 ms in the swing phase, after the demonstrator’s right heel touched the treadmill (Fig 1). The timing, number, and order of stimuli were controlled with a pulse control device (Pulse Timer Unit; Medical Try System Co., Tokyo, Japan) and control software (Pulse Timer II; Medical Try System Co., Tokyo, Japan).

### Measurement of imagery ability

On a separate day after the TMS measurements, each individual’s motor imagery ability was evaluated using a mental chronometry test. The time it took each participant to imagine walking, beginning from the initial step and stopping at the 20th step (20 steps), was measured by the participant him/herself using a stopwatch while in a standing position (imagined walking time). Participants were instructed to imagine a self-selected normal speed of walking 20 steps as if they were moving, but without making any actual hand/foot movements. Movements were thus imagined from a first-person perspective. No instructions were provided as to whether to keep the eyes open or closed, or which leg should be used as the starting leg. Actual walking time was also measured using the same walking task. The time it took each participant to walk 20 steps from a static standing position was measured by an experimenter using a stopwatch. Participants were instructed to walk at a self-selected normal speed and, as above, the starting leg was not specified. Each measurement was conducted one time. Since previous research has shown that the performance order of imagined and actual walking has no effect on the difference between the imagined and actual walking times [47], randomization was not considered to be warranted, and the measurement of the imagined walking time always preceded that of the actual walking time.

### Data analysis

For each participant, eight MEP waveforms in each condition were processed offline, and an averaged MEP waveform was obtained using an examination software (Multi Stim Tracer; Medical Try System Co., Tokyo, Japan). Since the averaged MEP waveforms in the TA and SOL muscles were predominantly polyphasic, the areas under the curve of averaged MEPs (MEP areas) were used as the main outcome measure of the current study, in accordance with a previous study by Bakker et al. [48]. The area under the MEP curve was measured [22,49] using the same software, and representative values in individual conditions were calculated for further analyses.

To examine whether an observer’s visual experience and/or intention to imitate would affect the alterations of corticospinal excitability in the TA and SOL muscles, MEP areas in the stance or swing condition were divided by MEP areas in the baseline condition and expressed as relative MEP (R-MEP) areas. The R-MEP areas in individual groups were then calculated by averaging R-MEP areas in the stance and swing conditions together, represented as mean R-MEP areas. It is known that MEPs are modulated by background EMG activity [50]. Thus, to ensure that participants were completely relaxed and that no muscle contraction was generated during the TMS measurements, we assessed EMG activity for each subject and each muscle using integrated electromyograms, calculated from the rectified EMG data during the 50-ms period prior to TMS with the aforementioned software. The mean background EMG activity in each stimulus condition (baseline, stance, and swing conditions during PO trials [PO stance and PO swing], and stance and swing conditions during AO trials [AO stance and AO swing]) was also calculated. Since motor cortical activation during motor imagery, but not during action observation, is reportedly correlated with motor imagery ability [51], further analyses were performed to reveal if individual gait imagery ability affected corticospinal excitability during the AO trials in this study. As an index of gait imagery ability, the data obtained from the mental chronometry test were calculated as follows: gait imagery ability = |actual walking time–imagined walking time| / actual walking time × 100 [52]. We selected the data of participants who answered that they had explicitly imagined the demonstrator’s gait movements during AO trials, and each participant’s averaged R-MEP areas were used for the correlation analyses.

All statistical analyses were performed using IBM SPSS Statistics 22 (IBM SPSS Statistics Inc., Tokyo, Japan). A one-way repeated measures analysis of variance (ANOVA) with the factor of stimulus condition (baseline, PO stance, PO swing, AO stance, AO swing) was applied to confirm whether the alterations of corticospinal excitability in the TA and SOL muscles during gait observation were step cycle-dependent or not. When a significant main effect was identified, post-hoc tests (Bonferroni correction) for multiple comparisons were performed. The background EMG activity, calculated from the integrated electromyograms, during the five stimulus conditions was also analyzed using a one-way repeated measures ANOVA to rule out effects of background activity on the changes in MEP areas. Comparisons of characteristics between the visual experience and control groups, including mean age, mean TMS stimulation intensity, mean raw MEP areas in the TA and SOL muscles in the baseline condition, and mean gait imagery ability, were analyzed using independent samples t-tests. We then performed a three-way mixed model ANOVA to analyze the mean R-MEP areas in the TA and SOL muscles during the two observation conditions using group (visual experience group, control group) as the between-subjects variable and observation condition (PO trial, AO trial) and muscle (TA, SOL) as within-subjects variables. Furthermore, the correlation between gait imagery ability and the R-MEP area in each of the two muscles during the AO trials was analyzed using a Pearson’s product moment correlation coefficient. Unless otherwise stated, results are expressed as the mean ± standard error of the mean. The alpha level for statistical significance for all analyses was set to P = 0.05, and effect sizes were reported as partial eta squared values (η_p_^2^).

## Results

In eight participants, measurable MEP data could not be elicited from the TA and SOL muscles simultaneously, and some visible background electromyographic (EMG) activity was recorded in two participants during the AO trials. Thus, the data from these participants were excluded from further analysis. Ultimately, the MEP data from 26 participants (13 participants per group) were included in the final analyses.

### General effects of gait observation on corticospinal excitability in the TA and SOL muscles

**Fig 2:**
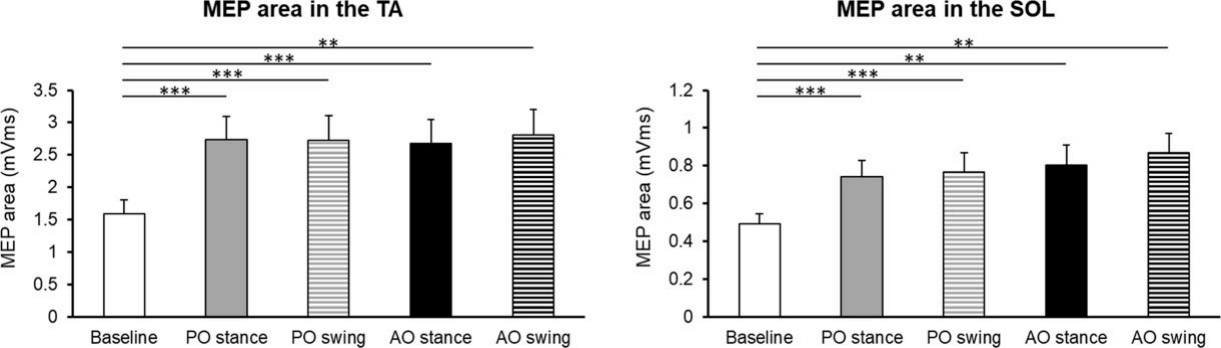
Motor-evoked potentials (MEPs) in the tibialis anterior (TA) and soleus (SOL) muscles during the rest and gait observation conditions. MEPs were recorded from the TA and SOL muscles during rest (baseline; observed static treadmill without the demonstrator), and during the midpoints of the stance phase (stance condition) and swing phase (swing condition) in both passive observation (PO; observed movements of the demonstrator’s lower legs) and active observation (AO; observed movements of the demonstrator’s lower legs with the intent to imitate) trials. The observation of periodic gait itself led to significant corticospinal facilitation in both muscles compared to baseline, irrespective of the step-cycle or observation condition. **P < 0.01, ***P < 0.001.

A one-way repeated measures ANOVA showed a significant main effect of stimulus condition on the MEP areas in the TA (F_2.673,66.823_ = 12.381, P < 0.001, η_p_^2^ = 0.331) and SOL (F_2.210,55.248_ = 11.877, P < 0.001, η_p_^2^ = 0.322) muscles (Fig 2). Post-hoc tests revealed that the MEP areas recorded from the TA and SOL muscles were significantly higher in the AO stance, AO swing, PO stance, and PO swing conditions during gait observation than in the baseline condition. However, no significant differences in the MEP areas were found among the other stimulus conditions. In contrast, no significant differences in background EMG data were identified in the TA (F_2.614,65.361_ = 1.015, P = 0.384) and SOL (F_2.910,72.758_ = 1.336, P = 0.270) muscles among the five stimulus conditions (Table 2), indicating that the modulation of MEP areas may have been induced not by alterations in slight background EMG signals but by the effects of gait observation.

**Table 2:** Mean background EMG activity (mVms) and SE from the TA and SOL muscles during each stimulus condition.

Muscle	Stimulus condition				
	Baseline	PO stance	PO swing	AO stance	AO swing
**TA**	0.134 (0.005)	0.133 (0.005)	0.133 (0.005)	0.134 (0.006)	0.136 (0.006)
**SOL**	0.176 (0.010)	0.172 (0.011)	0.171 (0.011)	0.173 (0.012)	0.174 (0.012)

EMG, electromyographic; SE, standard error; TA, tibialis anterior; SOL, soleus; PO, passive observation; AO, active observation.

### Effects of visual experience and/or different observation conditions on corticospinal excitability during gait observation

**Fig 3:**
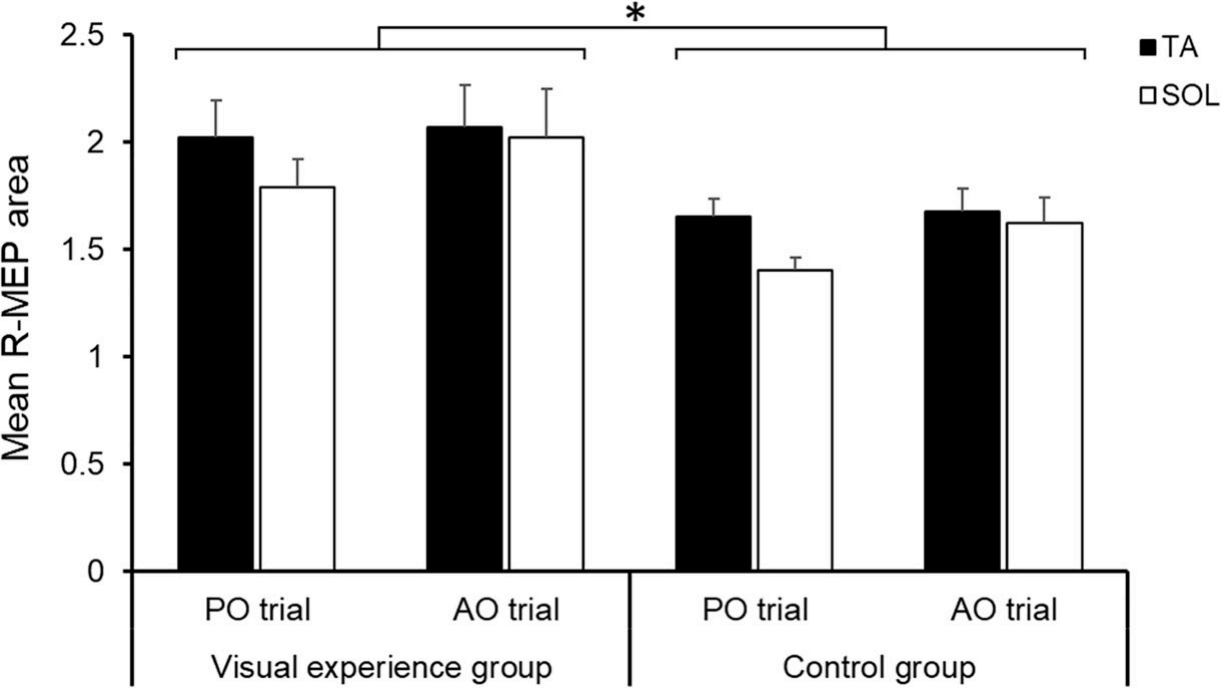
Effects of the observer’s visual experience or intention to imitate on the mean relative motor-evoked potentials (R-MEPs). MEPs were obtained from the tibialis anterior (TA) and soleus (SOL) muscles during passive observation (PO) and active observation (AO) trials in observers with (visual experience group) or without (control group) visual experience. The corticospinal facilitation effects in both muscles were enhanced by the observer’s experience with gait observation. *P < 0.05.

We found no significant differences between the visual experience and control groups in terms of mean age (t = 1.594, P = 0.124), mean TMS stimulation intensity (t = 0.115, P = 0.910), mean raw MEP areas in the TA (t = -0.189, P = 0.852) and SOL (t = -0.681, P = 0.503) muscles in the baseline condition, and mean gait imagery ability (t = 0.498, P = 0.623) (Table 1). Fig 3 displays the mean R-MEP areas in the TA and SOL muscles for the two observation conditions (PO and AO trials) in observers with or without experience in gait observation. The three-way mixed model ANOVA (group × observation condition × muscle) revealed a significant main effect of group (F_1,50_ = 5.274, P = 0.026, η_p_^2^ = 0.095), with the mean R-MEP area being significantly higher in the visual experience group than in the control group. However, we did not identify a significant three-way interaction (F_1,50_ = 0.007, P = 0.932), group × observation condition interaction (F_1,50_ = 0.015, P = 0.902), or group × muscle interaction (F_1,50_ = 0.006, P = 0.940). This indicates that individual visual experience independently affected corticospinal excitability irrespective of observation condition and facilitated it during both PO and AO trials. Moreover, the main effects of observation condition (F_1,50_ = 2.843, P = 0.098, η_p_^2^ = 0.054) and muscle (F_1,50_ = 3.469, P = 0.068, η_p_^2^ = 0.065) were not significant, nor was the observation condition × muscle interaction (F_1,50_ = 3.082, P = 0.085, η_p_^2^ = 0.058).

### Correlation between gait imagery ability and alterations in TA or SOL muscle corticospinal excitability

**Fig 4:**
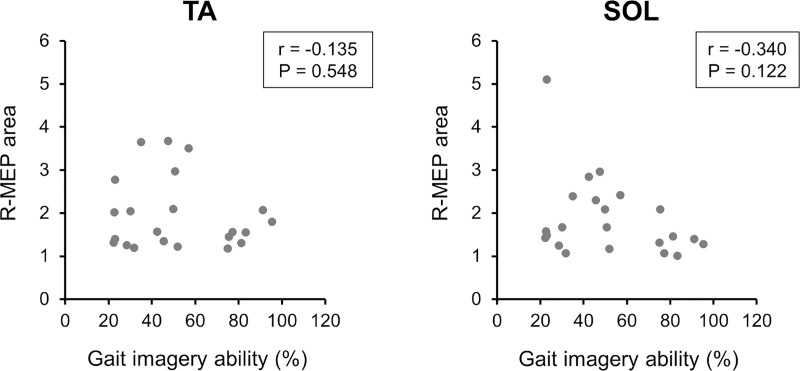
Correlation between gait imagery ability and alterations of corticospinal excitability in the tibialis anterior (TA) or soleus (SOL) muscle. The area under the MEP curve in the stance or swing condition was divided by that in the baseline condition, thus yielding the relative motor-evoked potential (R-MEP) area. No significant correlation was identified between gait imagery ability and the R-MEP area in the TA or SOL muscles.

Twenty-two of the 26 participants explicitly reported that they had imagined the observed demonstrator’s gait during AO trials. Thus, the data from these 22 individuals were selected to be used in the correlation analysis. The relationship between motor imagery ability, as assessed with the mental chronometry test, and corticospinal changes in the TA or SOL muscles, as reflected by the averaged R-MEP areas, is shown in Fig 4. Pearson’s test revealed no significant correlation between gait imagery ability and the R-MEP area in the TA (r = -0.135, P = 0.548) or SOL (r = -0.340, P = 0.122) muscles. These results suggest that the facilitations in the corticospinal tract to the TA and SOL muscles during gait observation were not related to the participants’ gait imagery ability.

## Discussion

### Characteristics of motor cortical activity during cyclic gait observation

Firstly, in the present study, we used single-pulse TMS to verify whether temporal and muscular observation/execution matching of neural activity in the motor cortex exists while observing periodic gait. Since it is well known that the flexor and extensor muscles in the lower limbs are activated in a reciprocal manner during steady gait, we selected the TA and SOL muscles as the target muscles to address this question. We found that gait observation significantly facilitated corticospinal excitability, as demonstrated by elevated MEPs in both the TA and SOL muscles during gait observation relative to baseline, irrespective of the step-cycle condition (stance or swing) and observation condition (PO or AO trials). During actual gait execution, the TA muscle is activated at the midpoint of the swing phase in the absence of SOL activity, whereas the SOL muscle is activated during the stance phase in the absence of TA activity [53]. Thus, our findings indicate that the motor resonance elicited by TMS during gait observation is incongruent with the changes in crural muscle activity that occur during actual gait and is independent of the step cycle, in agreement with previous research by Takahashi et al. [30].

One interpretation of this finding is that a functional role of the motor cortex throughout the step cycle may be the primary cause of the different alterations in corticospinal excitability that occur during gait observation vs. gait execution. As for the temporal muscle activity pattern during steady cyclic gait, it has been suggested that spinal neuronal networks, termed central pattern generators (CPGs), are involved in the generation of locomotor rhythms and patterns [54,55]. In fact, the presence of spinal CPGs in humans was ascertained via epidural stimulation of the lumber cord in individuals with complete spinal cord injury, whereby non-patterned stimulation of the lumber spinal cord was reported to induce step-like EMG activity [56]. Hence, spinal locomotor networks play a crucial role in the timing of motor bursts in leg muscles in a phase-dependent manner during cyclic gait execution, which may be associated with the constant corticospinal facilitation of gait-related muscles that we found in this study. Taken together, these results suggest that although cyclic gait observation itself facilitates the motor system, implementation of AOT using only steady gait may not promote the learning of gait patterns or muscle activity patterns in patients with gait disturbances in clinical settings.

### Effects of visual experience and/or intention to imitate on motor cortical activity during gait observation

Regarding the effects of visual experience on motor cortex activity, we found that corticospinal excitability changes were significantly higher in the visual experience group than in the control group during gait observation. Therefore, visual experience in observing gait, as well as the kinematic knowledge of gait shared by participants in the visual experience group, seems to modulate motor cortex circuits during gait observation. A previous study showed that motor circuits are activated more in individuals with visuomotor and visual expertise when they observe actions belonging to their domain of expertise than in individuals without such experience [18]. Moreover, even without motor experience, corticospinal excitability is enhanced as a function of an individual’s visual experience [57]. In a developmental study [58], stronger cortical activation was displayed in infants’ motor systems during the observation of naturally acquired actions, such as crawling, with which the infants were more familiar than with walking. Based on this study, we allocated coeval healthy individuals to the visual experience and control groups according to their major field of study at university. Motor experience with gait did not differ between the two groups and was controlled homogeneously in the current study. Our results thus suggest that the visual experience of gait observation itself enhances motor cortex facilitation during the observation of another individual’s gait.

Although the current study revealed a statistically significant main effect of group, the detected effect size of η_p_^2^ = 0.095 (f = 0.324) was smaller than the value (f = 0.595) that was used to determine the sample size. In the current experiment, participants were asked to focus their attention not on just a portion of the demonstrator’s leg joints, e.g., only the ankle joint, but on the lower legs as a whole, which allowed participants to freely observe the demonstrator’s gait in different ways. Additionally, human walking has the peculiar characteristic of being a complex lower limb movement that is principally generated by the coordination of bilateral hip, knee, and ankle joints. Thus, one possible reason for the small effect size may be the variation in observation sites and methods that observers used during gait observation. In support of this speculation, several recent TMS studies demonstrated that directing an observer’s visual attention to a fixed part of an observed movement or action-relevant object enhances corticospinal excitability during the observation of thumb abduction/adduction movements [59] and hand grasping actions [60–62]. Given these definite effects of an observer’s overt attention on motor resonance, it can be assumed that the results obtained in this study were also substantially affected by the participants’ attention. One reasonable interpretation of the higher motor cortical activation that we identified in individuals with vs. without visual gait experience is that since participants in the visual experience group had kinematic knowledge of gait (e.g., regarding the functional role of crural muscles or the importance of ankle movement during gait), their volitional and selective attention may have been more frequently directed to ankle joint movements during gait observation, relative to participants in the control group.

Notably, although visual experience has been shown to affect corticospinal excitability in muscles related to the observed movements [57], we did not find a statistically significant interaction effect between group and muscle in this study, suggesting that visual gait experience does not modulate the excitability of gait-related muscles when observing cyclic gait. A study on the involvement of the primary motor cortex in actual human gait demonstrated that TMS-evoked MEPs in the SOL muscles are reduced during the stance phase, while MEPs in the inactive TA muscle are enhanced compared to those elicited during voluntary ankle plantar flexion [43]. Based on these results, the authors proposed that the corticospinal tract is more closely linked to segmental motor circuits controlling the TA muscle than to those controlling the SOL muscle. Our study showed that excitability changes in the corticospinal tract to the TA muscle were moderately enhanced compared to those to the SOL muscle during mere gait observation. Therefore, our results appear to be comparable with the functional activity observed in the motor cortex during actual periodic gait [43].

We had speculated that differences in motor imagery ability between individuals with and without visual experience might modulate motor cortex activity during the two observation conditions (PO and AO trials). However, visual experience did not have any effect on corticospinal excitability during the two observation conditions. Williams et al. [51] demonstrated that despite the lack of a relationship between alterations in corticospinal excitability during action observation and imagery ability, there was a significant correlation between corticospinal excitability during motor imagery and imagery ability. Our complementary results showing no significant difference in gait imagery ability between the two visual experience groups and no significant correlation between gait imagery ability and excitability changes in the corticospinal tract to the TA or SOL support, at least in part, the absence of a group × observation condition interaction effect in this study.

To clarify the effects of an observer’s intent to imitate on motor cortex activity, we analyzed AO and PO trials as two distinct observation conditions. Previous research has demonstrated that observing a hand/finger movement with the intention to imitate elicits greater modulations in motor areas than passively observing the same action [34,36]. However, although AO trials tended to generate a stronger corticospinal facilitation than PO trials in our study, the differences were not significant.

A previous study by Hardwick et al. [63] regarding the influence of intent to imitate during action observation on motor cortex activity used TMS and reported the contradictory finding that passive, but not active, observation of grasping and finger abduction-adduction significantly facilitates corticospinal excitability compared to a control condition (observation of a fixation cross). To explain this finding, the authors proposed that an inhibitory mechanism acting on the corticospinal system prevents the immediate overt imitation of the observed action. In contrast, Wright et al. [38] described a motor facilitation effect during observation with the intent to imitate, as a strategy employed by participants to learn movement sequences. Additionally, the observation of a complex finger-sequence task with the intent to imitate was reported to produce higher corticospinal excitability than the observation of a simple task [36]. In both of these previous studies [36,38], participants were required to learn, through observation, a movement sequence that was part of a complex task, rather than simple hand/finger movements as in the study by Hardwick et al. [63]. Although normal walking is a complex movement task achieved by the coordination of multiple joints and muscles, the observation of human walking cannot be regarded as a complex observation task for learning new movement sequences, due to the automatic processing of walking movements. Therefore, in our study, we did not find any overt motor facilitation effects of the intent to imitate on motor cortex activity.

The current study has several limitations. Although prior research has revealed that an observer’s visual attention can facilitate corticospinal excitability [59–62], we could not distinguish the effects of visual attention from those of visual experience on primary motor cortex activity in this study. In addition, several brain regions have been reported to be involved in mere action observation and action observation with the intent to imitate [12,64], and changes in corticospinal excitability during action observation would be directly affected by not only the modulation of the primary motor cortex and spinal cord but also the activity of other brain regions [65]. However, the actual activity of the whole brain was not examined; thus, it is unclear how either visual experience or intention to imitate affects various cortical regions and modulates motor circuits during gait observation. To better understand the critical elements underlying and promoting the process of motor learning in AOT, further experiments are needed to clarify the correlation between visual experience and attention, the combined or individual influence of these two factors on motor cortex activity during action observation, and the effects of visual experience or the intent to imitate on the activity of the whole brain during gait observation.

## Conclusions

Here, corticospinal facilitation identified during cyclic gait observation was not tightly matched to the muscle activity patterns known to occur in gait-related lower leg muscles during actual gait, and was consistent throughout the entire step cycle. Moreover, the present study demonstrates, for the first time, that this motor cortical resonance to gait observation is enhanced by an individual’s visual experience (including kinematic knowledge of gait), even in the absence of differences in visuomotor experience, such as with human walking.

## Supporting information

S1 Table Individual data of MEP area. (XLSX)

S2 Table Individual data of background EMG activity. (XLSX)

S3 Table Individual data of imagery ability. (XLSX)

## Abbreviations

ANOVA: analysis of variance
AO: active observation
AOT: action observation therapy
CPG: central pattern generator
EMG: electromyographic
fMRI: functional magnetic resonance imaging
MEP: motor-evoked potential
PO: passive observation
R-MEP: relative motor-evoked potential
SOL: soleus
TA: tibialis anterior
TMS: transcranial magnetic stimulation
